# Data from a pooled post hoc analysis of 14 placebo-controlled, dapagliflozin treatment studies in patients with type 2 diabetes with and without anemia at baseline

**DOI:** 10.1016/j.dib.2021.107237

**Published:** 2021-06-21

**Authors:** Bergur V. Stefánsson, Hiddo J.L. Heerspink, David C. Wheeler, C. David Sjöström, Peter J. Greasley, Peter Sartipy, Valerie Cain, Ricardo Correa-Rotter

**Affiliations:** a Late-stage Development, Cardiovascular, Renal and Metabolism, BioPharmaceuticals R&D, AstraZeneca, Gothenburg, Sweden; b Clinical Pharmacy and Pharmacology, University Medical Center Groningen, Groningen, The Netherlands; c George Institute for Global Health, Sydney, Australia; d Department of Renal Medicine, University College London, London, United Kingdom; e Research and Early Development, Cardiovascular, Renal and Metabolism, BioPharmaceuticals R&D, AstraZeneca, Gothenburg, Sweden; f Systems Biology Research Center, School of Bioscience, University of Skövde, Skövde, Sweden; g Bogier Clinical and IT Solutions, Raleigh, North Carolina, United States; h Instituto Nacional de Ciencias Médicas y Nutrición Salvador Zubirán, Mexico City, Mexico

**Keywords:** Anemia, Chronic kidney disease, Dapagliflozin, eGFR, Hematocrit, Hemoglobin, SGLT2 inhibitor, Type 2 diabetes

## Abstract

Dapagliflozin is a highly selective sodium-glucose cotransporter 2 inhibitor associated with stabilization of estimated glomerular filtration rate (eGFR); reductions in glycated hemoglobin (HbA1c), systolic blood pressure, body weight, and albuminuria; and a small and consistent increase in hematocrit [1], [2], [3], [4]. This data set is based on the associated article [5] analyzing data from 5325 patients with type 2 diabetes from 14 placebo-controlled, phase 3 (one phase 2/3), double-blind dapagliflozin treatment studies of 24–104 weeks’ duration. Data on dapagliflozin's effects (vs. placebo) on hemoglobin (Hb), hematocrit, serum albumin, serum total protein concentrations, urine albumin/creatinine ratio, eGFR, heart rate, blood pressure, body weight, and safety in patients with type 2 diabetes with and without anemia were pooled and analyzed. Patients were divided into two groups according to baseline Hb levels: anemia (Hb <13 g/dL in men and <12 g/dL in women) and no anemia. Some biomarkers associated with erythropoiesis and the presence of anemia, such as iron, transferrin, ferritin, reticulocytes, and hepcidin, were not included in the original studies and therefore data for these biomarkers were not available. Descriptive statistics were used for baseline characteristics and safety data and a longitudinal repeated-measures mixed model for efficacy data. Changes in Hb concentrations were evaluated, and the proportion of patients with baseline anemia who were no longer anemic at week 24 was determined, as was the occurrence of polycythemia (Hb >16.5 g/dL in men and >16.0 g/dL in women). Because anemia commonly occurs in patients with diabetes and chronic kidney disease [6], the data can be of value to further analyze trends in relevant physiological and pathophysiological parameters.

**Specifications Table**

**Table:** 

Subject	Endocrinology, Diabetes and Metabolism				
Specific subject area	Type 2 diabetes, Anemia				
Type of data	Tables				
How data were acquired	Data from the 14 studies were originally acquired by patient and investigator report and laboratory tests				
Data format	Raw Analyzed				
Parameters for data collection	Demographic and baseline characteristics, hematocrit, Hb, serum albumin and serum total protein concentrations, urine albumin/creatinine ratio, eGFR, systolic and diastolic blood pressure, heart rate, body weight, and adverse events in patients with type 2 diabetes with baseline anemia (Hb <13 g/dL in men and <12 g/dL in women) and no anemia.				
Description of data collection	Data were collected according to standard clinical trial procedures from 14 clinical studies that were part of the dapagliflozin clinical development program.				
Data source location	Primary data sources: Please refer to Supplementary Table 1 of the associated article, which is reproduced here: Dapagliflozin clinical trials included in the pooled analysis (redrawn from Supplementary Table 1 of the related research article)				
	Study Name ClinicalTrials.gov Identifier	Phase	Title	Treatment arms	Treatment duration

MB102-013 NCT00528372	3	A Multicenter, Randomized, Double-Blind, Placebo-Controlled, Parallel Group, Phase 3 Trial to Evaluate the Safety and Efficacy of Dapagliflozin as Monotherapy in Subjects With Type 2 Diabetes Who Have Inadequate Glycemic Control With Diet and Exercise	Dapagliflozin (2.5, 5, or 10 mg) and placebo	102 weeks
(*continued on next page*)

**Table:** 

	MB102-014 NCT00528879	3	A Phase III Study of BMS-514148 (Dapagliflozin) in Patients With Type 2 Diabetes Who Are Not Well Controlled on Metformin Alone	Dapagliflozin (2.5, 5, or 10 mg) and placebo	102 weeks
	MB102-029 (CKD) NCT00663260	3	Trial to Evaluate the Glycemic Efficacy, Renal Safety, Pharmacokinetics, and Pharmacodynamics of Dapagliflozin in Subjects With Type 2 Diabetes Mellitus and Moderate Renal Impairment Who Have Inadequate Glycemic Control	Dapagliflozin (5 or 10 mg) and placebo	104 weeks
	MB102-030 NCT00683878	3	A Multicenter, Randomized, Double-Blind, Placebo-Controlled, Parallel Group, Phase 3 Trial to Evaluate the Safety and Efficacy of Dapagliflozin in Combination With Thiazolidinedione Therapy in Subjects With Type 2 Diabetes Who Have Inadequate Glycemic Control on Thiazolidinedione Therapy Alone	Dapagliflozin 5 mg + pioglitazone Dapagliflozin 10 mg + pioglitazone Pioglitazone + placebo	48 weeks
	MB102-034 NCT00859898	3	A Multicenter, Randomized, Double-Blind, Active Controlled, Parallel Group, Phase 3 Trial to Evaluate the Safety and Efficacy of Dapagliflozin 10 mg in Combination With Metformin as Initial Therapy as Compared With Dapagliflozin 10 mg Monotherapy and Metformin Monotherapy in Subjects With Type 2 Diabetes Who Have Inadequate Glycemic Control	Dapagliflozin 10 mg + metformin XR Dapagliflozin 10 mg + placebo Metformin XR + placebo	24 weeks
	D1690C00005 NCT00680745	3	A 24-Week Randomized, Double-blind, Parallel-Group, Multicentre, Placebo-Controlled Phase III Study to Evaluate the Efficacy and Safety of Dapagliflozin in Combination With Glimepiride (a Sulphonylurea) in Subjects With Type 2 Diabetes Who Have Inadequate Glycaemic Control on Glimepiride Therapy Alone	Dapagliflozin (2.5, 5, or 10 mg) + glimepiride Placebo + glimepiride	48 weeks
	D1690C00006 NCT00673231	3	A 24-Week International, Randomized, Parallel-Group, Double-Blind, Placebo Controlled Phase III Study With a 80-Week Extension Period to Evaluate the Efficacy and Safety of Dapagliflozin Therapy When Added to the Therapy of Patients With Type 2 Diabetes With Inadequate Glycaemic Control on Insulin	Dapagliflozin (2.5, 5, or 10 mg) and placebo	104 weeks
	D1690C00010 NCT00984867	3	A 24-Week, Multicentre, Randomised, Double-Blind, Placebo-Controlled, International Phase 3 Study With a 24-Week Extension Period to Evaluate the Safety and Efficacy of Dapagliflozin 10 mg QD in Patients With Type 2 Diabetes Who Have Inadequate Glycaemic Control on a DPP-4 Inhibitor (Sitagliptin) Alone or in Combination With Metformin	Dapagliflozin 10 mg and placebo	48 weeks
	D1690C00012 NCT00855166	3	A 24-Week, Multicentre, International, Double-blind, Randomized, Parallel-Group, Placebo-Controlled, Phase III Study With a 78-Week Extension Period to Evaluate the Effect of Dapagliflozin in Combination With Metformin on Body Weight in Subjects With Type 2 Diabetes Mellitus Who Have Inadequate Glycemic Control on Metformin Alone	Dapagliflozin 10 mg + metformin Placebo + metformin	102 weeks
	D1690C00018 NCT01031680	3	A 24-Week, Multicentre, Randomised, Double-Blind, Age-Stratified, Placebo Controlled Phase III Study With an 80-Week Extension Period to Evaluate the Efficacy and Safety of Dapagliflozin 10 mg Once Daily in Patients With Type 2 Diabetes, Cardiovascular Disease and Hypertension, Who Exhibit Inadequate Glycaemic Control on Usual Care	Dapagliflozin 10 mg and placebo	104 weeks
	D1690C00019 NCT01042977	3	A 24-Week, Multicentre, Randomised, Double-Blind, Age-Stratified, Placebo Controlled Phase III Study With an 80-Week Extension Period to Evaluate the Efficacy and Safety of Dapagliflozin 10 mg Once Daily in Patients With Type 2 Diabetes and Cardiovascular Disease, Who Exhibit Inadequate Glycaemic Control on Usual Care	Dapagliflozin 10 mg and placebo	104 weeks
	D1690C00023 (DELIGHT) NCT02547935	2/3	An Exploratory Phase II/III, Randomized, Double-Blind, Placebo Controlled, Parallel Design Study to Evaluate the Efficacy, Safety and Pharmacodynamics of Dapagliflozin and Dapagliflozin in Combination With Saxagliptin in CKD Patients With Type 2 Diabetes Mellitus and Albuminuria Treated With ACEi or ARB	Dapagliflozin 10 mg Dapagliflozin 10 mg + saxagliptin Placebo	24 weeks
	D1690C00024 (DERIVE) NCT02413398	3	A Multicenter, Double-Blind, Placebo-Controlled, Parallel Group, Randomized, Phase III Study to Evaluate the Glycemic Efficacy and Renal Safety of Dapagliflozin in Patients With Type 2 Diabetes Mellitus and Moderate Renal Impairment (CKD 3A) Who Have Inadequate Glycemic Control	Dapagliflozin 10 mg and placebo	24 weeks
	D1692C00006 (Japan) NCT01294423	3	A 24-Week Randomised, Double-Blind, Parallel-Group, Multi-Centre, Placebo-Controlled Phase III Trial to Evaluate the Efficacy and Safety of Dapagliflozin as Monotherapy in Japanese Subjects With Type 2 Diabetes Who Have Inadequate Glycemic Control With Diet and Exercise	Dapagliflozin (5 or 10 mg) and placebo	24 weeks
Data accessibility	Repository name: AstraZeneca Clinical Trials Website. Readers can access the data set via AstraZeneca's website (in accordance with AstraZeneca's data sharing policy described at https://astrazenecagrouptrials.pharmacm.com/ST/Submission/Disclosure) upon request and approval of a committee. Direct URL to data: https://astrazenecagrouptrials.pharmacm.com/ST/Submission/Search				
(*continued on next page*)

**Table:** 

Related research article	The data presented in this article are related to [5]. B.V. Stefánsson, H.J.L. Heerspink, D.C. Wheeler, C.D. Sjöström, P.J. Greasley, P. Sartipy, V. Cain, R. Correa-Rotter. Correction of anemia by dapagliflozin in patients with type 2 diabetes, J Diabetes Complications. 34 (2020) 107729, 10.1016/j.jdiacomp.2020.107729.				

## Value of the Data

• Our data report on a variety of safety and efficacy parameters in patients with type 2 diabetes (*N* = 5325) with and without anemia from 14 placebo-controlled, phase 3 (one phase 2/3), double-blind studies of dapagliflozin treatment over 24–104 weeks. The data contribute to understand the effects of dapagliflozin treatment in patients with type 2 diabetes with and without anemia.
• These data may be useful to diabetologists, endocrinologists, nephrologists, hematologists, cardiologists, and patients.
• The dapagliflozin treatment data included here expand upon those reported in the associated research article and could be used to interrogate Hb, blood pressure, body weight, and other physiological trends over time. The data could be used to inform the design or interpretation of other studies or analyses of sodium-glucose cotransporter 2 inhibitors.
• A strength of this data set is that it contains pooled data from multiple placebo-controlled, double-blind studies, providing an overview of a large patient population, including in patients with eGFR <60 mL/min/1.73 m^2^.
• The data provide a detailed longitudinal picture of adverse events in patients with type 2 diabetes with and without anemia undergoing dapagliflozin treatment.

## 1 Data Description

• **Table Set 1**: Demographic and Baseline Disease Characteristics Summary From 14 Placebo-Controlled Studies With at Least 24 Weeks of Treatment, by Anemia Subgroup
• **Table Set 2**: Adverse Events Suggestive of Renal Impairment, Urinary Tract Infection, and Volume Depletion, by Preferred Term, 24-Week Double-blind Treatment Period in Patients With eGFR <60 mL/min/1.73 m^2^, by Anemia Subgroup
• **Table Set 3**: Summary of Urine Albumin-to-Creatinine Ratio (mg/g) at Baseline, Hb (g/dL) at Week 24, and Adverse Events Suggestive of Renal Impairment, Urinary Tract Infection, and Volume Depletion, by Preferred Term, by Anemia Subgroup
• **Table Set 4**: Hb (g/dL) Adjusted Percent Change From Baseline Longitudinal Repeated Measures Analysis, 24-Week Double-blind Treatment Period in Patients With eGFR <60 mL/min/1.73 m^2^, by Anemia Subgroup
• **Table Set 5**: Serum Albumin (g/dL) and Serum Total Protein (g/dL) Longitudinal Repeated Measures Analysis, 24-Week Double-blind Treatment Period, by Anemia Subgroup
• **Table Set 6**: Demographic and Baseline Disease Characteristics Summary, and Multiple Efficacy and Safety Results From 14 Placebo-Controlled Studies With at Least 24 Weeks of Treatment, in Patients With eGFR <60 mL/min/1.73 m^2^, by Anemia Subgroup Complete list of tables within each set is provided in Appendix 1.

Efficacy data include Hb, HbA1c, hematocrit, total body weight, sitting systolic and diastolic blood pressure, sitting heart rate, eGFR, urine albumin/creatinine ratio, and the proportion of patients with changes in anemia status from baseline to week 24. Safety data include overall summary of adverse events and adverse events in select system organ classes.

## 2 Experimental Design, Materials and Methods

Pooled data were from 14 phase 3 (one phase 2/3), double-blind, placebo-controlled studies of 24–104 weeks’ duration that included dapagliflozin 10 mg/day monotherapy in patients with type 2 diabetes (*N* = 5325). None of the studies were primarily designed to examine the effect of dapagliflozin on anemia.

The population was divided according to baseline Hb concentrations into anemia (Hb <13 g/dL in men and <12 g/dL in women) and no-anemia groups based on criteria defined by the World Health Organization. The studies’ protocols did not include specific restrictions or recommendations regarding supplemental iron or diet. Data on race were available and are reported but data on patient ethnicity were not recorded across all trial sites and are thus not reported.

The change in Hb concentrations over 24 weeks in patients receiving dapagliflozin or placebo in the anemia and no-anemia groups was evaluated. As part of the standard clinical trial safety assessment during the individual studies, blood samples were collected and analyzed at central laboratories. Hb concentrations were measured at baseline and at weeks 4, 8, 12, 16, 20, and 24. Changes in Hb concentrations in patients with or without baseline anemia were evaluated, and the proportion of patients with baseline anemia who were no longer anemic at week 24 was determined.

Changes from baseline to week 24 in eGFR (calculated using the Modification of Diet in Renal Disease Study equation), serum albumin, blood pressure, and body weight were also evaluated. Safety outcomes included the occurrence of adverse events and serious adverse events, including those of special interest (renal impairment, urinary tract infection, and volume depletion). Occurrence of polycythemia (Hb >16.5 g/dL in men and >16.0 g/dL in women) was also evaluated.

Descriptive statistics were used for presenting baseline characteristics and safety data. For efficacy parameters, we derived the mean changes from baseline values and 95% confidence intervals using a longitudinal repeated-measures mixed model with fixed terms for study, treatment, group, treatment-by-group interaction, week, week-by-group interaction, week-by-treatment interaction, and treatment-by-week-by-group interaction, along with the fixed covariates of baseline, baseline-by-week interaction, and baseline-by-study interaction. Degrees of freedom in the mixed model were approximated by the Kenward-Roger method. If the model(s) did not converge, the models were either re-run using the Kenward-Roger method with the baseline-by-week and baseline-by-study terms removed or the Satterthwaite approximation was used. SAS® version 9.4 (SAS Institute Inc.) was used for statistical analyses.

## Ethics Statement

All protocols from the studies were approved by the relevant institutional review board/ethics committee. Written informed consent was provided by all enrolled patients. The studies were conducted in accordance with the principles of the Declaration of Helsinki.

## Declaration of Competing Interest

B.V.S., C.D.S., P.J.G., and P.S. are employees and shareholders of AstraZeneca. H.J.L.H. is a consultant to AbbVie, Astellas, AstraZeneca, Boehringer Ingelheim, Janssen, and ZS-Pharma (honoraria were paid to his employer). D.C.W. has received consultancy fees or honoraria from Amgen, AstraZeneca, Bayer, Boehringer Ingelheim, GlaxoSmithKline, Janssen, Napp, Mundipharma, Pharmacosmos, Reata, and Vifor Fresenius. V.C. is a former employee of AstraZeneca and owns AstraZeneca stock. R.C.R. has received honoraria from AbbVie, AstraZeneca, GlaxoSmithKline, and Boehringer Ingelheim, and has lectured for Amgen, Janssen, Takeda, AstraZeneca, Boehringer Ingelheim, and Roche.

## Appendix 1

• **Table 1.1:** Demographic and Baseline Characteristics, Full Analysis Set, Anemia – Pages 1–6
• **Table 1.2:** Demographic and Baseline Characteristics, Full Analysis Set, No Anemia – Pages 7–12
• **Table 1.3:** Baseline Medications and Disease History, Full Analysis Set, Anemia – Page 13
• **Table 1.4:** Baseline Medications and Disease History, Full Analysis Set, No Anemia – Page 14
• **Table 1.5:** Longitudinal Repeated Measures Analysis of Hemoglobin (24-week Short Term, Double-blind Treatment Period), Safety Analysis Set, Anemia – Pages 15–17
• **Table 1.6:** Longitudinal Repeated Measures Analysis of Hemoglobin (24-week Short Term, Double-blind Treatment Period), Safety Analysis Set, No Anemia – Pages 18–20
• **Table 1.7:** Longitudinal Repeated Measures Analysis of HbA1c (24-week Short Term, Double-blind Treatment Period), Full Analysis Set, Anemia – Pages 21–23
• **Table 1.8:** Longitudinal Repeated Measures Analysis of HbA1c (24-week Short Term, Double-blind Treatment Period), Full Analysis Set, No Anemia – Pages 24–26
• **Table 1.9:** Longitudinal Repeated Measures Analysis of Hematocrit (24-week Short Term, Double-blind Treatment Period), Safety Analysis Set, Anemia – Pages 27–29
• **Table 1.10:** Longitudinal Repeated Measures Analysis of Hematocrit (24-week Short Term, Double-blind Treatment Period), Safety Analysis Set, No Anemia – Pages 30–32
• **Table 1.11:** Longitudinal Repeated Measures Analysis of Total Body Weight (24-week Short Term, Double-blind Treatment Period), Full Analysis Set, Anemia – Pages 33–35
• **Table 1.12:** Longitudinal Repeated Measures Analysis of Total Body Weight (24-week Short Term, Double-blind Treatment Period), Full Analysis Set, No Anemia – Pages 36–38
• **Table 1.13:** Longitudinal Repeated Measures Analysis of Sitting Systolic Blood Pressure (24-week Short Term, Double-blind Treatment Period), Full Analysis Set, Anemia – Pages 39–41
• **Table 1.14:** Longitudinal Repeated Measures Analysis of Sitting Systolic Blood Pressure (24-week Short Term, Double-blind Treatment Period), Full Analysis Set, No Anemia – Pages 42–44
• **Table 1.15:** Longitudinal Repeated Measures Analysis of Sitting Diastolic Blood Pressure (24-week Short Term, Double-blind Treatment Period), Full Analysis Set, Anemia – Pages 45–47
• **Table 1.16:** Longitudinal Repeated Measures Analysis of Sitting Diastolic Blood Pressure (24-week Short Term, Double-blind Treatment Period), Full Analysis Set, No Anemia – Pages 48–50
• **Table 1.17:** Longitudinal Repeated Measures Analysis of Sitting Heart Rate (24-week Short Term, Double-blind Treatment Period), Full Analysis Set, Anemia – Pages 51–53
• **Table 1.18:** Longitudinal Repeated Measures Analysis of Sitting Heart Rate (24-week Short Term, Double-blind Treatment Period), Full Analysis Set, No Anemia – Pages 54–56
• **Table 1.19:** Longitudinal Repeated Measures Analysis of eGFR (24-week Short Term, Double-blind Treatment Period), Full Analysis Set, Anemia – Pages 57–59
• **Table 1.20:** Longitudinal Repeated Measures Analysis of eGFR (24-week Short Term, Double-blind Treatment Period), Full Analysis Set, No Anemia – Pages 60–62
• **Table 1.21:** Longitudinal Repeated Measures Analysis of Urine Albumin/Creatinine Ratio (24-week Short Term, Double-blind Treatment Period), Full Analysis Set, Anemia – Pages 63–65
• **Table 1.22:** Longitudinal Repeated Measures Analysis of Urine Albumin/Creatinine Ratio (24-week Short Term, Double-blind Treatment Period), Full Analysis Set, No Anemia – Pages 66–68
• **Table 1.23:** Proportion of Subjects with Changes in Anemia Status from Baseline to Week 24, Safety Analysis Set – Page 69
• **Table 1.24:** Overall Adverse Events (24-Week Short-term Double-blind Treatment Period), Safety Analysis Set, Anemia – Page 70
• **Table 1.25:** Overall Adverse Events (24-Week Short-term Double-blind Treatment Period), Safety Analysis Set, No Anemia – Page 71
• **Table 1.26:** Vascular Disorders, Blood and Lymphatic System Disorders, and Cardiac Disorders (24-week Short Term, Double-blind Treatment Period), Safety Analysis Set, Anemia – Pages 72–74
• **Table 1.27:** Vascular Disorders, Blood and Lymphatic System Disorders, and Cardiac Disorders (24-week Short Term, Double-blind Treatment Period), Safety Analysis Set, No Anemia – Pages 75–80
• **Table 2.1:** Adverse Events Suggestive of Renal Impairment by Preferred Term (24-week Short Term, Double-blind Treatment Period), Safety Analysis Set, Anemia – Page 2
• **Table 2.2:** Adverse Events Suggestive of Renal Impairment by Preferred Term (24-week Short Term, Double-blind Treatment Period), Safety Analysis Set, No Anemia – Page 3
• **Table 2.3:** Adverse Events Suggestive of Urinary Tract Infection by Preferred Term (24-week Short Term, Double-blind Treatment Period), Safety Analysis Set, Anemia – Page 4
• **Table 2.4:** Adverse Events Suggestive of Urinary Tract Infection by Preferred Term (24-week Short Term, Double-blind Treatment Period), Safety Analysis Set, No Anemia – Page 5
• **Table 2.5:** Adverse Events Suggestive of Volume Depletion by Preferred Term (24-week Short Term, Double-blind Treatment Period), Safety Analysis Set, Anemia – Page 6
• **Table 2.6:** Adverse Events Suggestive of Volume Depletion by Preferred Term (24-week Short Term, Double-blind Treatment Period), Safety Analysis Set, No Anemia – Page 7
• **Table 3.1:** Proportion of subjects with Urine Albumin/Creatinine (UACR) >30 mg/g at Baseline, Full Analysis Set, Anemia – Page 2
• **Table 3.2:** Proportion of subjects with Urine Albumin/Creatinine (UACR) >30 mg/g at Baseline, Full Analysis Set, No Anemia – Page 3
• **Table 3.3:** Summary Statistics for Hemoglobin at Week 24 (LOCF) in subjects with polycythemia at Week 24 (LOCF), Safety Analysis Set – Page 4
• **Table 3.4:** Adverse Events Suggestive of Renal Impairment by Preferred Term (24-week Short Term, Double-blind Treatment Period), Safety Analysis Set, Anemia – Page 5
• **Table 3.5:** Adverse Events Suggestive of Renal Impairment by Preferred Term (24-week Short Term, Double-blind Treatment Period), Safety Analysis Set, No Anemia – Page 6
• **Table 3.6:** Adverse Events Suggestive of Urinary Tract Infection by Preferred Term (24-week Short Term, Double-blind Treatment Period), Safety Analysis Set, Anemia – Page 7
• **Table 3.7:** Adverse Events Suggestive of Urinary Tract Infection by Preferred Term (24-week Short Term, Double-blind Treatment Period), Safety Analysis Set, No Anemia – Page 8
• **Table 3.8:** Adverse Events Suggestive of Volume Depletion by Preferred Term (24-week Short Term, Double-blind Treatment Period), Safety Analysis Set, Anemia – Page 9
• **Table 3.9:** Adverse Events Suggestive of Volume Depletion by Preferred Term (24-week Short Term, Double-blind Treatment Period), Safety Analysis Set, No Anemia – Page 10
• **Table 4.1:** Hemoglobin (g/dL) Adjusted Percent Change from Baseline Longitudinal Repeated Measures Analysis (24-Week Short-term Double-blind Treatment Period), Safety Analysis Set, Anemia – Pages 2–4
• **Table 4.2:** Hemoglobin (g/dL) Adjusted Percent Change from Baseline Longitudinal Repeated Measures Analysis (24-Week Short-term Double-blind Treatment Period), Safety Analysis Set, No Anemia – Pages 5–7
• **Table 5.1:** Longitudinal Repeated Measures Analysis of Serum Albumin (24-week Short Term, Double-blind Treatment Period), Safety Analysis Set, Anemia – Pages 2–4
• **Table 5.2:** Longitudinal Repeated Measures Analysis of Serum Albumin (24-week Short Term, Double-blind Treatment Period), Safety Analysis Set, No Anemia – Pages 5–7
• **Table 5.3:** Longitudinal Repeated Measures Analysis of Serum Total Protein (24-week Short Term, Double-blind Treatment Period), Safety Analysis Set, Anemia – Pages 8–10
• **Table 5.4:** Longitudinal Repeated Measures Analysis of Serum Total Protein (24-week Short Term, Double-blind Treatment Period), Safety Analysis Set, No Anemia – Pages 11–13
• **Table 6.1:** Demographic and Baseline Characteristics, Full Analysis Set, Anemia – Pages 2–8
• **Table 6.2:** Demographic and Baseline Characteristics, Full Analysis Set, No Anemia – Pages 9–15
• **Table 6.3:** Baseline Medications and Disease History, Full Analysis Set, Anemia – Pages 16–17
• **Table 6.4:** Baseline Medications and Disease History, Full Analysis Set, No Anemia – Pages 18–19
• **Table 6.5:** Longitudinal Repeated Measures Analysis of Hemoglobin (24-Week Short-term Double-blind Treatment Period), Safety Analysis Set, Anemia – Pages 20–22
• **Table 6.6:** Longitudinal Repeated Measures Analysis of Hemoglobin (24-Week Short-term Double-blind Treatment Period), Safety Analysis Set, No Anemia – Pages 23–25
• **Table 6.7:** Longitudinal Repeated Measures Analysis of HbA1c (24-Week Short-term Double-blind Treatment Period), Full Analysis Set, Anemia – Pages 26–28
• **Table 6.8:** Longitudinal Repeated Measures Analysis of HbA1c (24-Week Short-term Double-blind Treatment Period), Full Analysis Set, No Anemia – Pages 29–31
• **Table 6.9:** Longitudinal Repeated Measures Analysis of Hematocrit (24-Week Short-term Double-blind Treatment Period), Safety Analysis Set, Anemia – Pages 32–34
• **Table 6.10:** Longitudinal Repeated Measures Analysis of Hematocrit (24-Week Short-term Double-blind Treatment Period), Safety Analysis Set, No Anemia – Pages 35–37
• **Table 6.11:** Longitudinal Repeated Measures Analysis of Total Body Weight (24-Week Short-term Double-blind Treatment Period), Full Analysis Set, Anemia – Pages 38–40
• **Table 6.12:** Longitudinal Repeated Measures Analysis of Total Body Weight (24-Week Short-term Double-blind Treatment Period), Full Analysis Set, No Anemia – Pages 41–43
• **Table 6.13:** Longitudinal Repeated Measures Analysis of Sitting Systolic Blood Pressure (24-Week Short-term Double-blind Treatment Period), Full Analysis Set, Anemia – Pages 44–46
• **Table 6.14:** Longitudinal Repeated Measures Analysis of Sitting Systolic Blood Pressure (24-Week Short-term Double-blind Treatment Period), Full Analysis Set, No Anemia – Pages 47–49
• **Table 6.15:** Longitudinal Repeated Measures Analysis of Sitting Diastolic Blood Pressure (24-Week Short-term Double-blind Treatment Period), Full Analysis Set, Anemia – Pages 50–52
• **Table 6.16:** Longitudinal Repeated Measures Analysis of Sitting Diastolic Blood Pressure (24-Week Short-term Double-blind Treatment Period), Full Analysis Set, No Anemia – Pages 53–55
• **Table 6.17:** Longitudinal Repeated Measures Analysis of Sitting Heart Rate (24-Week Short-term Double-blind Treatment Period), Full Analysis Set, Anemia – Pages 56–58
• **Table 6.18:** Longitudinal Repeated Measures Analysis of Sitting Heart Rate (24-Week Short-term Double-blind Treatment Period), Full Analysis Set, No Anemia – Pages 59–61
• **Table 6.19:** Longitudinal Repeated Measures Analysis of eGFR (24-Week Short-term Double-blind Treatment Period), Full Analysis Set, Anemia – Pages 62–64
• **Table 6.20:** Longitudinal Repeated Measures Analysis of eGFR (24-Week Short-term Double-blind Treatment Period), Full Analysis Set, No Anemia – Pages 65–67
• **Table 6.21:** Longitudinal Repeated Measures Analysis of Urine Albumin/Creatinine Ratio (24-Week Short-term Double-blind Treatment Period), Full Analysis Set, Anemia – Pages 68–70
• **Table 6.22:** Longitudinal Repeated Measures Analysis of Urine Albumin/Creatinine Ratio (24-Week Short-term Double-blind Treatment Period), Full Analysis Set, No Anemia – Pages 71–73
• **Table 6.23:** Proportion of Subjects with Changes in Anemia Status from Baseline to Week 24 (24-Week Short-term Double-blind Treatment Period), Safety Analysis Set – Page 74
• **Table 6.24:** Overall Adverse Events (24-Week Short-term Double-blind Treatment Period), Safety Analysis Set, Anemia – Page 75
• **Table 6.25:** Overall Adverse Events (24-Week Short-term Double-blind Treatment Period), Safety Analysis Set, No Anemia – Page 76
• **Table 6.26:** Vascular Disorders, Blood and Lymphatic System Disorders, and Cardiac Disorders (24-Week Short-term Double-blind Treatment Period), Safety Analysis Set, Anemia – Pages 77–79
• **Table 6.27:** Vascular Disorders, Blood and Lymphatic System Disorders, and Cardiac Disorders (24-Week Short-term Double-blind Treatment Period), Safety Analysis Set, No Anemia – Pages 80–84
